# Supplementary figures and images for: Efficacy assessment of a novel endolysin PlyAZ3aT for the treatment of ceftriaxone-resistant pneumococcal meningitis in an infant rat model
Source: PLoS One. 2022 Apr 26;17(4):e0266928. doi: 10.1371/journal.pone.0266928 (PMC9041855; doi:10.1371/journal.pone.0266928)

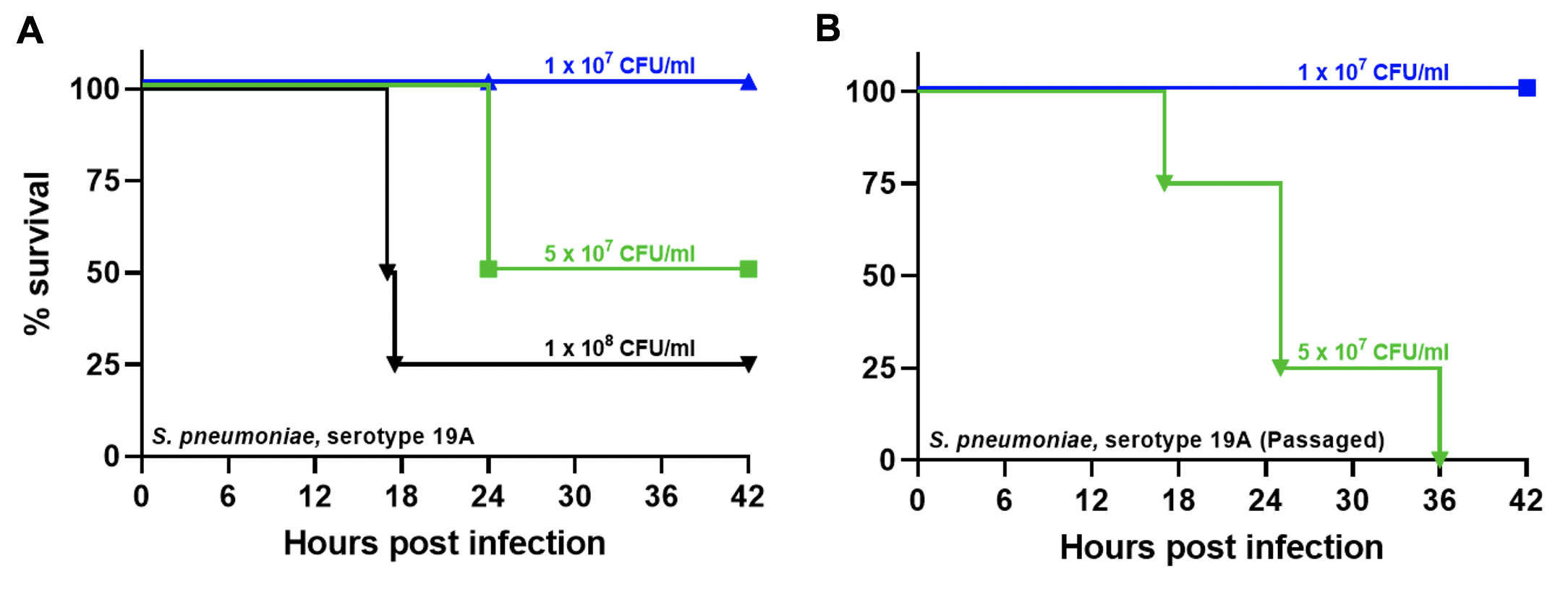

Supplement: S1 Fig — Kaplan-Meier survival curves for LD90 finding of the new strain (A) before and (B) after 3 passages in infant rats. 10 μl of the corresponding inoculum were injected. (TIF) [file pone.0266928.s001.tif]

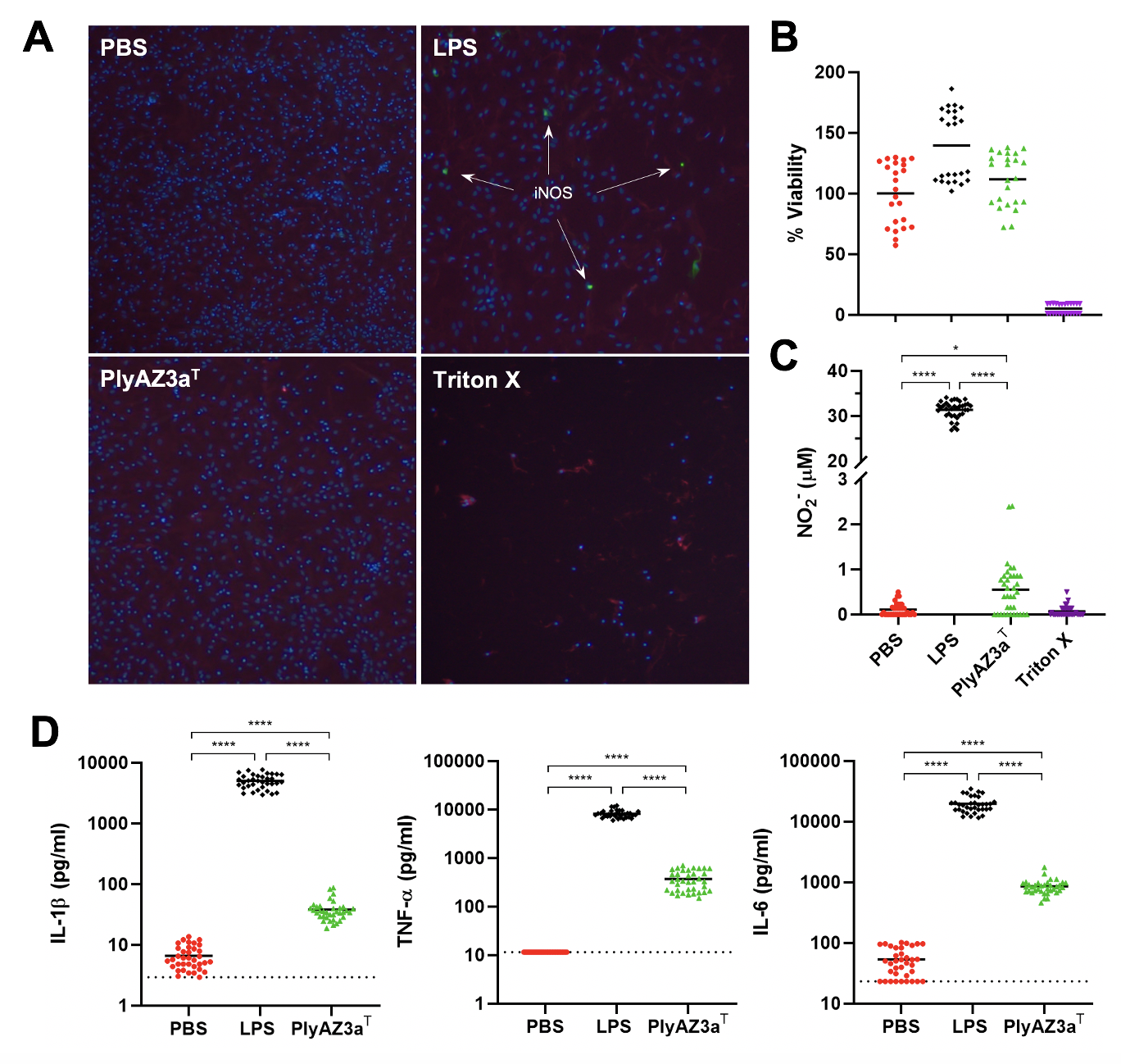

Supplement: S2 Fig — Cells were exposed for 24 h to either PBS (negative control), LPS (positive control), PlyAZ3aT or TritonX (membrane disrupting agent). (A) Immunohistological staining in astroglial cell cultures, iNOS is stained in green (anti-iNOS), astroglia is stained in red (anti-GFAP) and cell nuclei in blue (4′,6-Diamidin-2-phenylindole, DAPI). (B) Viability of cells was confirmed by using an XTT-assay (the mean of PBS treated cells is defined as 100%). (C) Production of NO2- measured as an index for nitric oxide (NO) release. (D) Levels of IL-1β, TNF-α and IL-6. Data are presented as mean ± standard deviation. Statistical differences between groups were assessed using Kruskal-Wallis test with Dunn’s multiple comparisons; *p<0.05, **** p<0.0001. (TIF) [file pone.0266928.s002.tif]

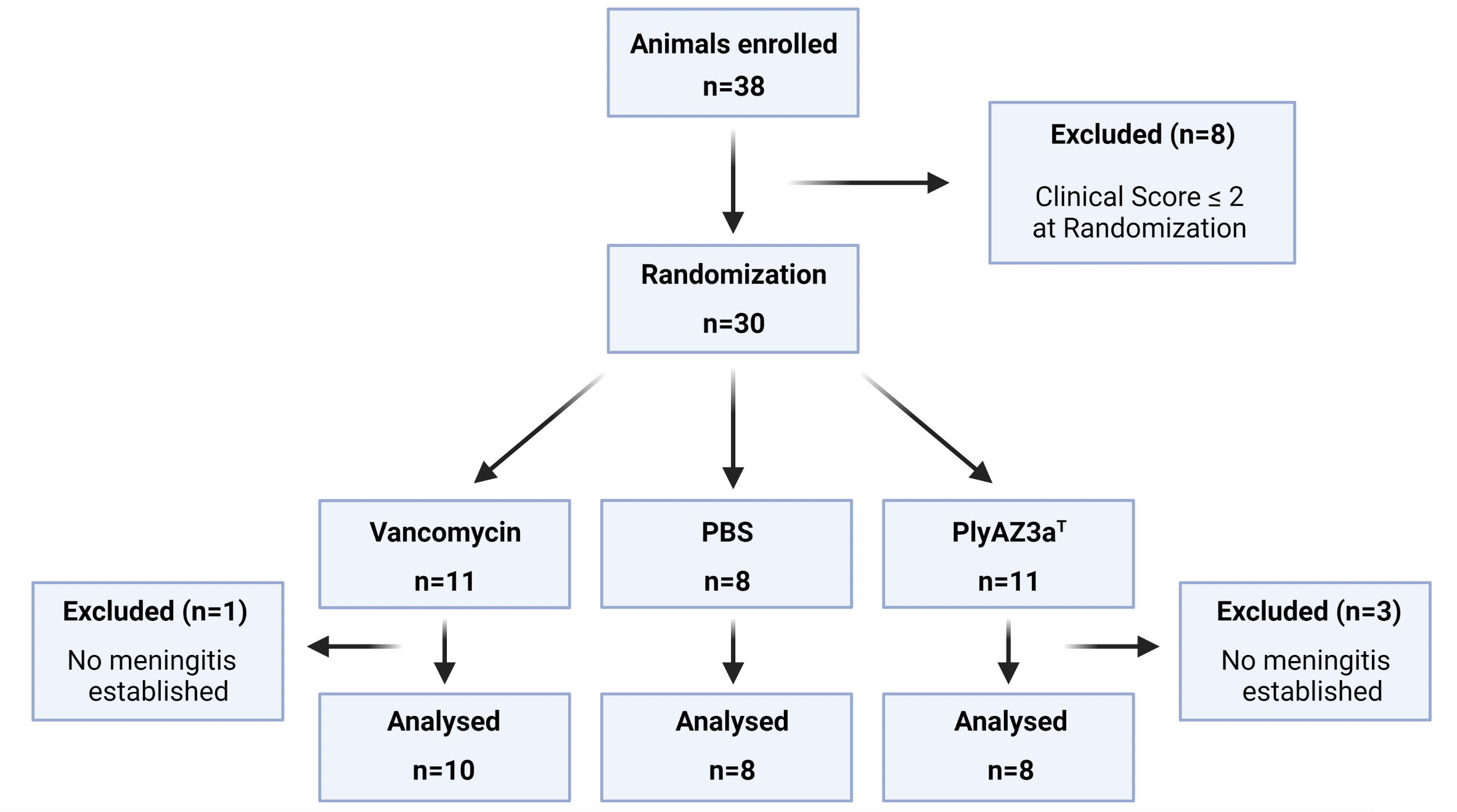

Supplement: S3 Fig — (TIF) [file pone.0266928.s003.tif]

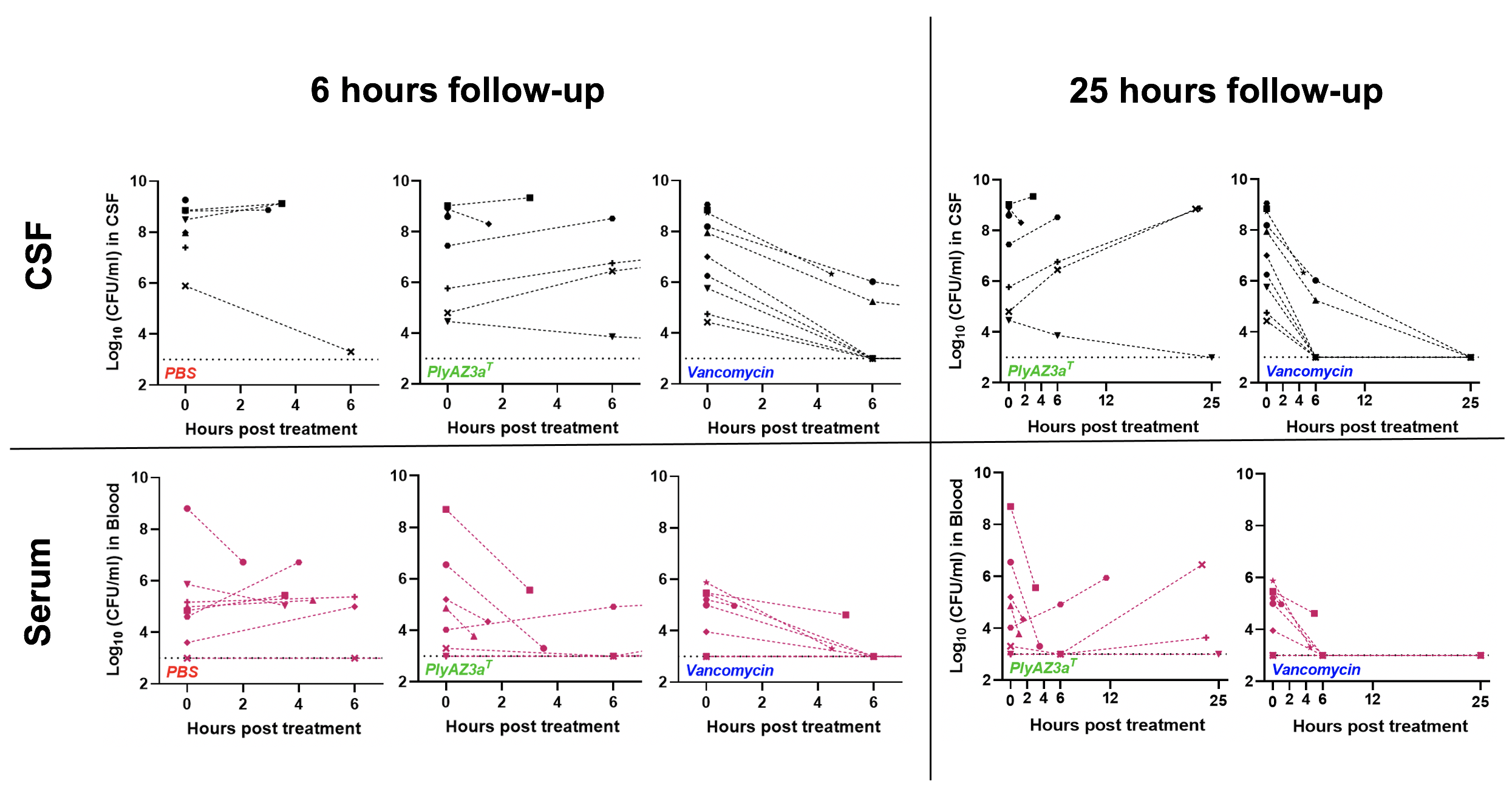

Supplement: S4 Fig — Tracking of bacterial loads displayed for the first six hours or until the end of the experiment (25 hours post treatment). Dots connected with lines represent repeated sampling in one single animal. CFU, colony forming unit. (TIF) [file pone.0266928.s004.tif]

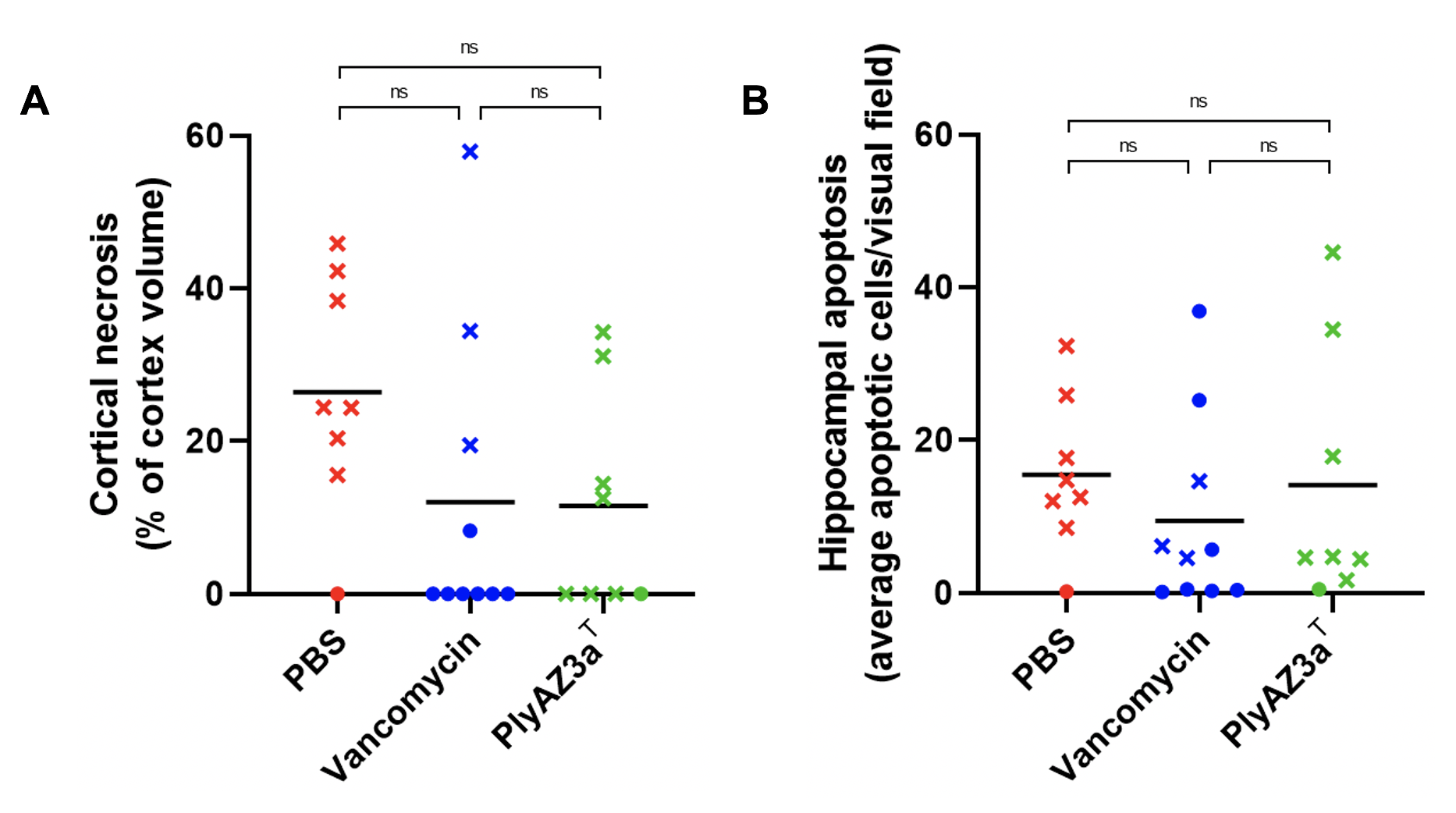

Supplement: S5 Fig — (A) Comparison in percentage of necrotizing cortex between groups and (B) number of apoptotic cells in the hippocampus. Animals reaching the end of the 42 hours trial are represented by closed circles and succumbed prematurely to the infection by crosses. Statistical significance was assessed using Kruskal-Wallis test with Dunn’s multiple comparisons, ns, not significant. (TIF) [file pone.0266928.s005.tif]

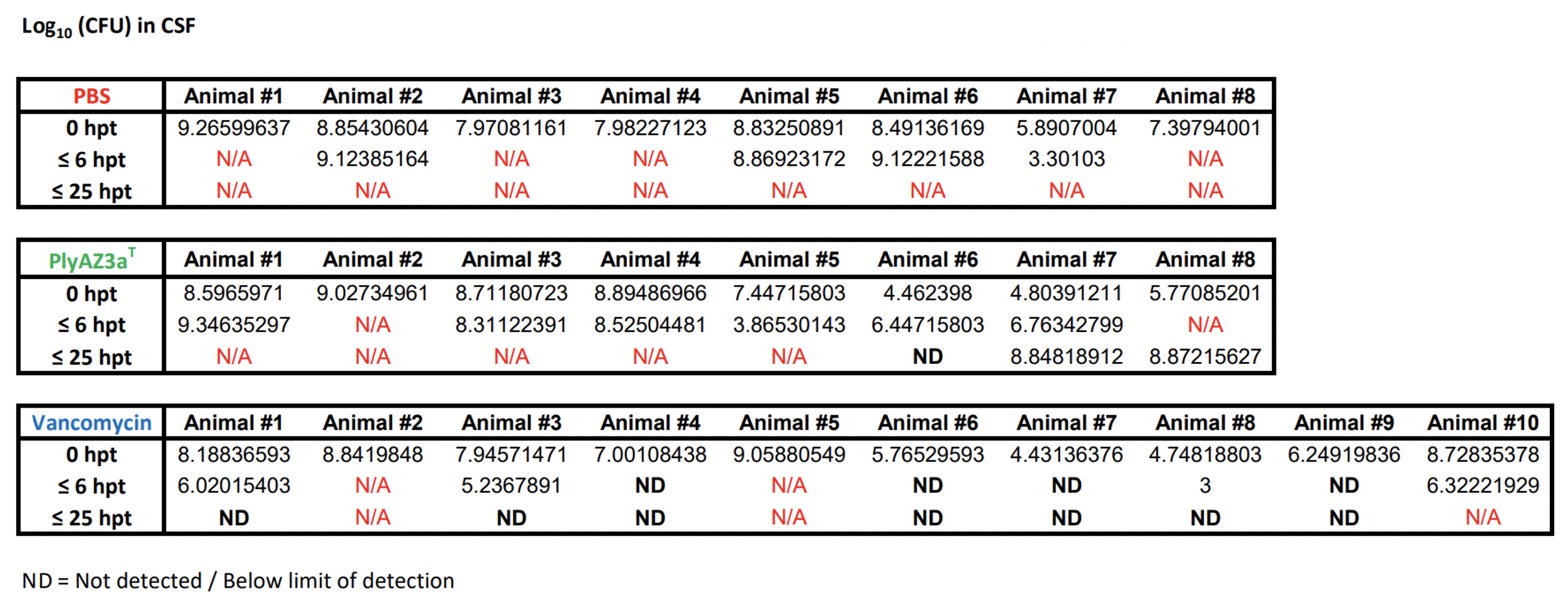

Supplement: S1 Table — Bacterial loads are displayed as Log10 (CFU). Hpt, hours post treatment. N/A, not available due to failed sampling. Limit of detection is 3 Log10. (TIFF) [file pone.0266928.s007.tiff]

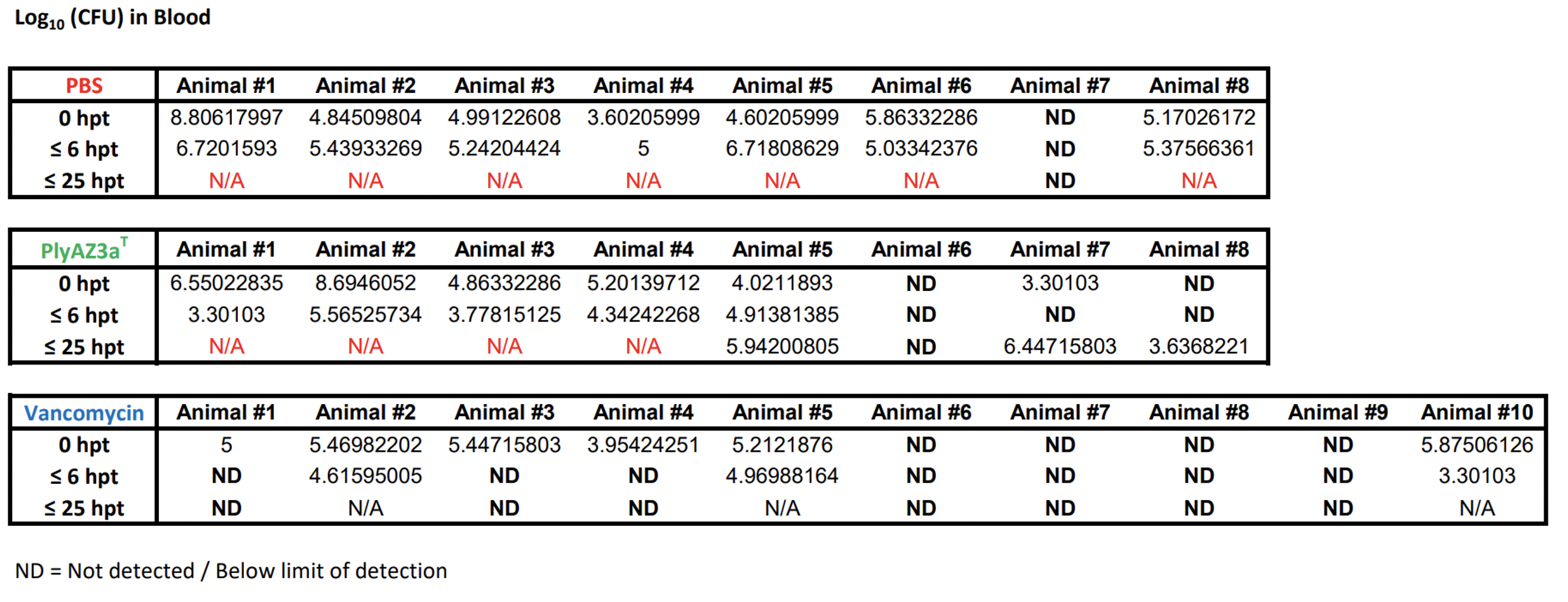

Supplement: S2 Table — Bacterial loads are displayed as Log10 (CFU). Hpt, hours post treatment. N/A, not available due to failed sampling. Limit of detection is 3 Log10. (TIFF) [file pone.0266928.s008.tiff]
