# Supplementary material for: Efficacy assessment of a novel endolysin PlyAZ3aT for the treatment of ceftriaxone-resistant pneumococcal meningitis in an infant rat model
Source: PLoS One. 2022 Apr 26;17(4):e0266928. doi: 10.1371/journal.pone.0266928 (PMC9041855; doi:10.1371/journal.pone.0266928)

Figure S6

Original Picture of Serum Westernblot in Figure 2B Without Labeling

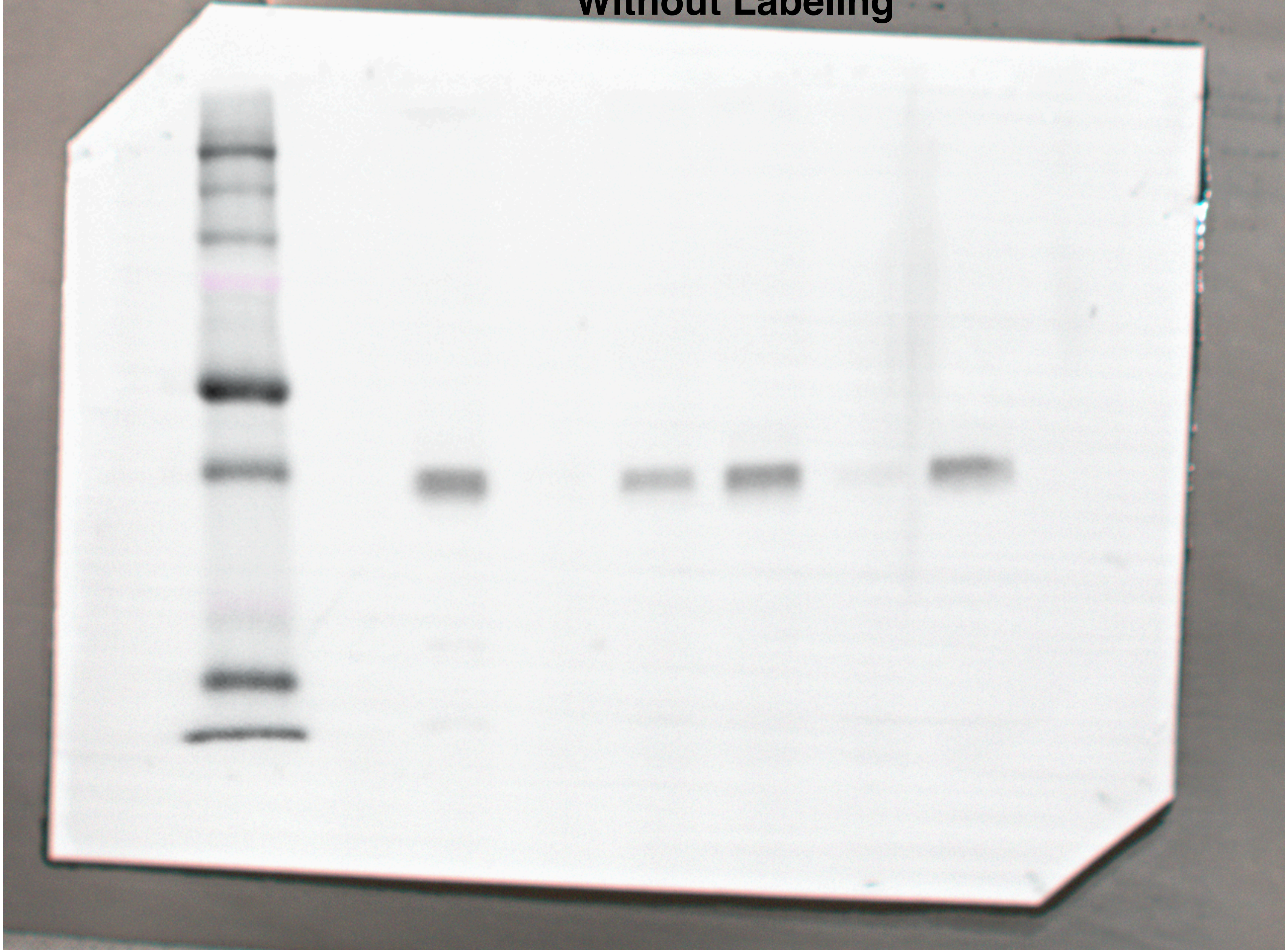

Figure S6

Original Picture of Serum Westernblot in Figure 2B With Labeling

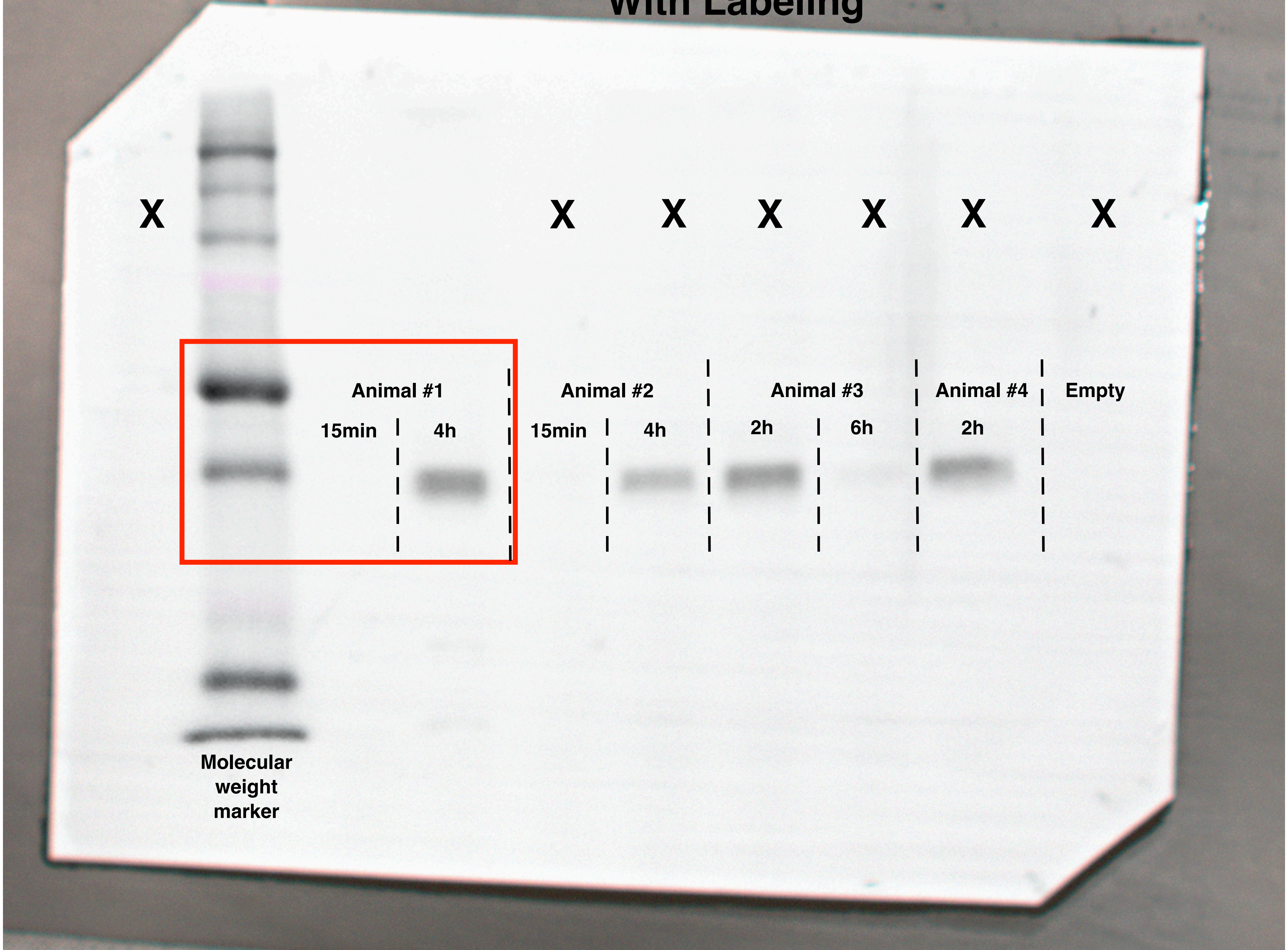

Figure S7 Original Picture of CSF Westernblot in Figure 2B Without Labeling

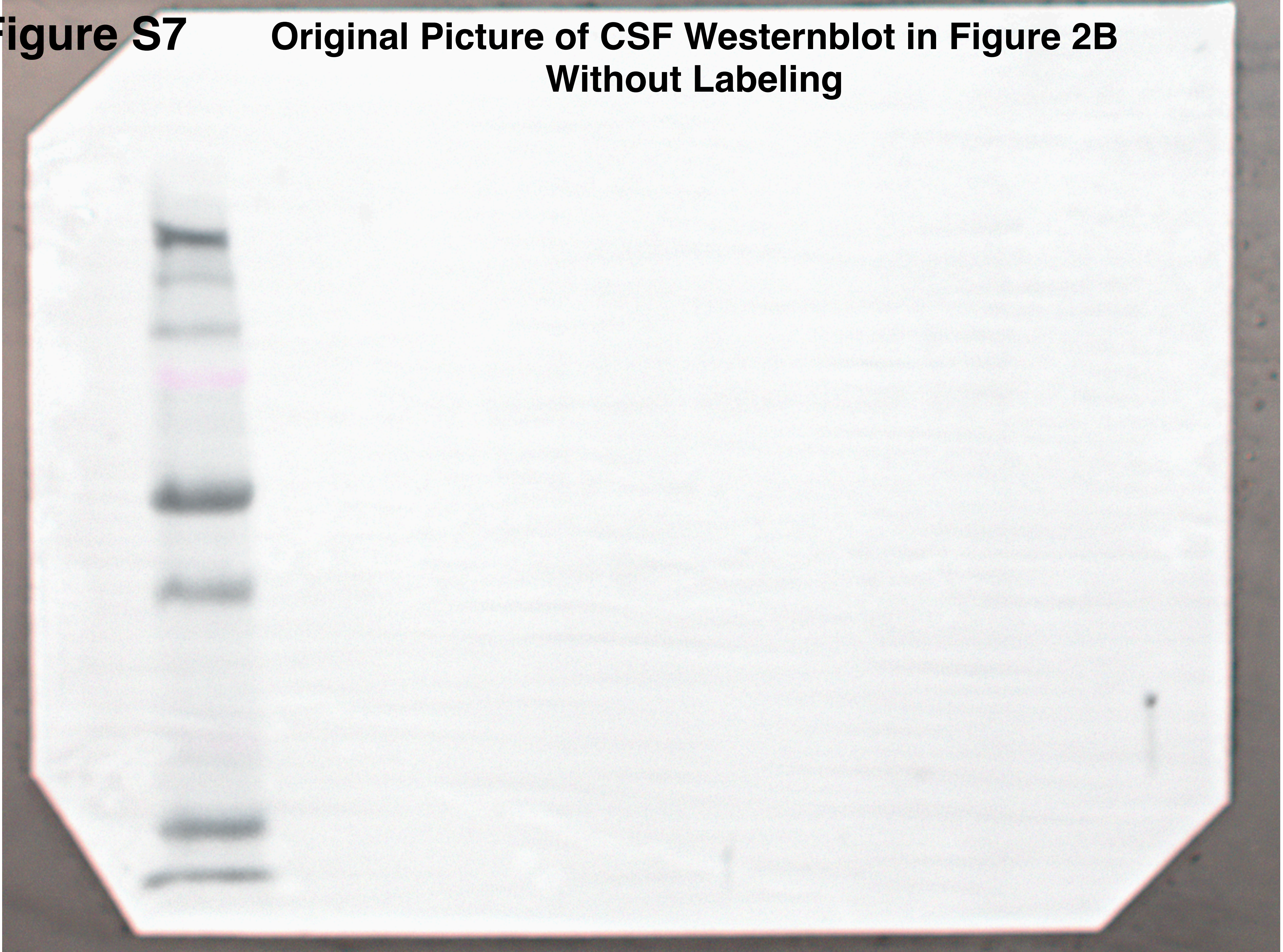

Figure S7

Original Picture of CSF Westernblot in Figure 2B Without Labeling

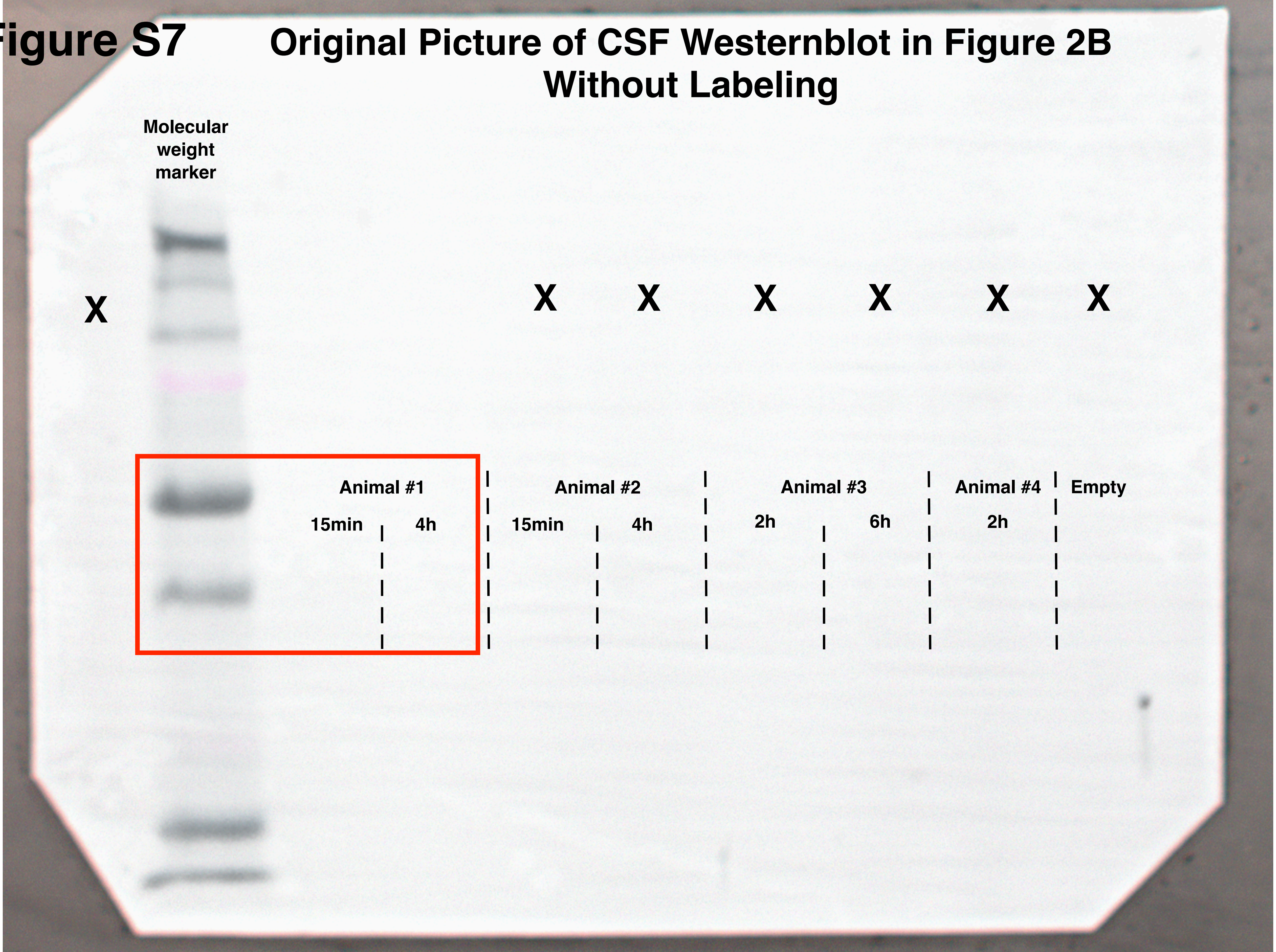

Supplement: S1 Raw images — The red rectangle corresponds to the section displayed in Fig 2B. Lanes not included in the final figure are marked with an “X”. (PDF) [file pone.0266928.s006.pdf]
